# Supplementary material for: Are carbon emissions trading and green financial instruments synergistic? -Comprehensive quantitative research based on content analysis
Source: PLoS One. 2024 Mar 7;19(3):e0298601. doi: 10.1371/journal.pone.0298601 (PMC10919601; doi:10.1371/journal.pone.0298601)
Supplement: S1 Data — (ZIP) [file pone.0298601.s001.zip › Data/地级市Z_F工作报告.zip!/╔╜╢1⁄2/╚╒╒╒╩╨/└┤╫╘╣1⁄2╓┌║┼í╛┬φ┐╦╩2╛▌═°í┐_2017.docx]

各位代表：

现在，我代表市人民政府向大会报告工作，请予审议，并请市政协各位委员提出意见。

一、本届政府工作回顾

过去的五年，是日照发展不平凡的五年。五年来，我们在省委、省政府和市委的坚强领导下，紧紧依靠全市人民，依靠市人大、市政协的监督支持，坚持稳中求进、改革创新，勇于担当、积极作为，在全面建成小康社会进程中迈出坚实步伐。

——综合实力迈上新台阶。2016年全市实现地区生产总值1802.5亿元，年均增长9.6%。一般公共预算收入128.7亿元，年均增长12.3%。规模以上工业增加值604.7亿元，年均增长10.5%。社会消费品零售总额660.1亿元，年均增长12.1%。固定资产投资五年累计完成6231.8亿元，年均增长16.6%。三次产业比例由2011年的8.9：54.4：36.7调整为8.1：47.3：44.6。威亚发动机三四工厂、派沃泰变速箱一二工厂、日钢ESP生产线、亚太森博液体包装纸等项目相继投产。山钢日照精品钢项目完成投资201.1亿元，一期一步工程今年8月投产。投资200亿元的清洁能源项目开工建设。

——基础设施建设取得新进展。日照机场2015年12月建成通航，已开通北上广等重点城市16个通达航班，年吞吐量突破40万人次。青日连铁路顺利开工，瓦日铁路建成通车，胶新铁路电气化改造投入运营，鲁南高铁日照段获批建设，“三纵四横”铁路交通网即将形成。长深高速莒县段、疏港高速建成通车，潍日高速开工建设。613省道、山海路、潮石路改造工程竣工，迎宾路快捷路改造、奎山综合客运站等一批重点工程开工建设。新建港口泊位12个，新增通过能力3935万吨。完成各类水利工程6600余处，沭水东调、龙潭沟水库等水利工程建成启用。城市日供水保障能力达到65万立方米。城市综合管廊、供电、供热、供气等基础设施不断完善。

——改革开放实现新突破。理顺了园区和招引体制机制，职员制、绩效工资制改革顺利推进。新组建了水务、公交、财金、交通发展、土地储备、海洋文旅等10家国有投融资平台，国有企业达到16家。在全市一次性推开校长职级制和去行政化改革，配套推进了基础教育管理体制改革。在全省首推“六证合一、一照一码”企业登记制度。完成485个村居集体产权股份合作试点，完成2567个村居承包地确权登记颁证。行政管理体制、公用事业、旅游、医药卫生等领域改革均取得实质性突破。对外开放步伐不断加快，五年累计实际到账外资26.8亿美元。成功举办中国—中亚合作论坛、中国日照—澳大利亚联邦北领地经贸合作交流会。中韩（日照）产业园集聚韩资企业32家。中澳（日照）产业园启动建设。综合保税区申建顺利推进。获批山东省跨境电子商务综合试验区。日照港口岸扩大开放获国务院批准。

——城乡面貌发生新变化。深入开展创城突出问题综合整治，市区三轮车、四轮代步车、卧龙山违建、露天烧烤、街头市场、店外经营等城市乱象得到彻底治理。2015年创城测评成绩在全国100个参评地级市中名列榜首。坚持放权、压责、让利，完善城中村改造政策，拆迁改造村居107个、房屋5万余处，整治改造老旧小区113个。实施万平口改造提升等系列工程。开工建设东夷小镇、海洋馆、龙门崮、马亓山、屋楼崮等一批旅游项目。建成运营华润万象汇、苏宁广场等城市综合体。新增城市道路410公里、综合管廊47公里、人防工程49.8万平方米。扎实推进美丽乡村建设，完成农村环卫市场化改革，新增农村公路通车里程1751公里，农村改厕17.4万户。2016年底城乡环卫一体化、移风易俗群众满意度调查全省第一。全面推进水、大气污染治理，关停全部园外石材企业1226家，淘汰全部水泥立窑生产线，市区饮用水源地水质达标率100%，空气质量保持在全省第一方阵。成功创建全国生态保护与建设示范区。被确定为首批国家级海洋公园、国家级海洋生态文明建设示范区。

——民生事业实现新进步。各级财政民生支出累计突破600亿元，占公共财政支出比重由2012年的63%提高到80.3%。城乡居民人均可支配收入年均分别增长9.9%、11.4%。城镇新增就业24.2万人，农村劳动力转移就业14.3万人。完成库区移民扶持项目971个，改善13.4万移民生活条件。完成10.3万人脱贫任务。建立居民医保与职工医保转换机制，实现城乡居民基本养老、基本医疗保险制度全覆盖。城乡居民基础养老金、失业保险金分别达到100元/月、950元/月。城乡居民低保标准分别达到490元/月、3600元/年。居民医保补助提高到420元。新建、改扩建学校396所，整建制通过“全国义务教育发展基本均衡县”验收。建成启用市图书馆新馆。基层综合性文化服务中心覆盖率80%以上，被文化部确定为全国示范地区。沂蒙画派跻身全国十大知名画派。在全省率先实现国家基本药物制度基层全覆盖，启动城市公立医院改革，实现药品“零差率”销售。被列为全省首批食品安全创建城市。全民健身活动蓬勃开展，新建农村公共体育健身设施1800余处。新建改造大型商业设施、社区便民消费网点900处。新投放新能源公交车100台。实现全国双拥模范城“五连冠”。连续两届荣获全国社会治安综合治理优秀市。完成首部《日照市志》（地级市）总纂稿。民族宗教、外事侨务、对台事务、打私、仲裁、档案、气象、红十字、妇女儿童、老龄、残疾人、慈善等事业健康发展。

各位代表，奋斗伴随艰辛，成绩来之不易。五年来，特别是2015年以来，我们坚持事不避难、勇于担当，面对经济下行、贸易融资风险等多重困难和压力，相继推出了“突破园区、聚力招引”、“五抓”组合拳、化解贸易融资风险、重点领域改革、旅游富市、林水会战、创城攻坚、效能建设、突破县域经济等一系列重大举措。我们把政府自身建设摆在突出位置，压减市级行政审批事项301项，严厉查处不担当、不作为、慢作为、乱作为行为。我们自觉接受人大、政协监督，累计办复人大代表建议790件、政协委员提案1580件。2016年，全市地区生产总值增长8.1%，一般公共预算收入同口径增长10.6%，均居全省第二位；固定资产投资增长13.5%，规模以上工业增加值增长8.6%，分别居全省第三位、第四位。在2016年度全省经济社会发展综合考核中，我市群众满意度从过去的全省下游跃居全省前列，其中干部表现全省第一。

过去五年取得的成绩，是省委、省政府和市委正确领导的结果，是各级人大、政协、社会各界有效监督和大力支持的结果，是全市人民同心同德、团结拼搏的结果。在此，我代表市人民政府，向全市人民、各界人士，向驻日照人民解放军指战员、武警官兵、公安干警，向关心支持日照发展的海内外朋友，表示衷心感谢并致以崇高敬意！

各位代表，肯定成绩的同时，我们也清醒地看到，发展中还存在不少困难和问题。经济发展不充分，优势和潜力还远远没有发挥出来。对外开放程度低，县域经济薄弱，培育新的经济增长点和转型升级任务艰巨。工业产业链条短、产品附加值低、创新能力弱。农业现代化水平不高，农产品品牌建设力度不够。民营经济和现代服务业发展层次低。园区基础设施建设滞后、承载能力不足，人才和技术支撑能力弱。教育、医疗、养老等基本公共服务供给不足、分配不均。资源环境承载压力大，水、气、土壤、城乡环境治理，与群众期盼还有差距。城市精细化管理水平亟待提高。一些部门单位懒政、怠政和不作为、乱作为的现象仍然存在，营商环境有待进一步改善，等等。这些问题，我们将坚持标本兼治，采取有力措施，认真加以解决。

二、今后五年的总体思路和奋斗目标

刚刚胜利闭幕的市第十三次党代会，科学描绘了日照发展的宏伟蓝图，对今后五年全市经济社会发展作出了全面部署。未来五年，是日照发展非常关键的五年，新一届市政府承担着重大历史使命。我们将认真贯彻落实市第十三次党代会战略部署，按照“一个目标、三个导向、五大战略”的总体思路，坚持以人民为中心的发展思想，紧紧围绕“追赶”“摘帽”目标，坚持问题导向、目标导向、用人导向，大力实施生态立市、工业强市、旅游富市、开放活市、人才兴市战略，提前全面建成小康社会，加快建设美丽富饶、生态宜居、充满活力的新日照。主要预期目标是：

让经济更加繁荣。到2019年，全市地区生产总值争取在全省前进1个位次；到2020年，全市人均生产总值达到全省平均水平，甩掉沿海欠发达帽子；到2021年，实现“双增双突破”，即：地区生产总值、一般公共预算收入比2016年分别增加1000亿元和100亿元，规模以上工业增加值突破1000亿元，五年固定资产投资突破10000亿元。“两区”达到全国百强县水平、“两县”达到全省中游水平，日照经济技术开发区经济发展进入全省前五位，日照高新区、岚山经济开发区创建为国家级园区，山海天旅游度假区创建为国家级旅游度假区，莒县经济开发区、市北经济开发区进入全省30强。

让人民更加幸福。坚持发展成果由人民共享，建立财政民生投入稳定增长机制。加快教育现代化进程，建成教育强市。建立覆盖城乡居民的基本医疗卫生制度。实现基层综合性文化服务中心全覆盖。建成覆盖城乡居民的社会保障体系和公共法律服务体系。城镇新增就业14.4万人。城市日供水保障能力110万立方米。农村饮水集中供水率达到80%以上。实行扶贫线与农村低保线“两线合一”，实现城乡低保标准一体化。2018年实现300个贫困村、44129名贫困人口全部脱贫。

让城市更加美丽。加快推进生态修复、城市修补，完善居民出行、购物、就学、就医等公共服务设施，倡树文明风尚，把城市和乡村打造得更靓丽、更宜居。全市常住人口城镇化率达到63.5%。城市建成区绿地率达到43.8%。完成主城区124个城中村改造任务。建设省级美丽乡村达标村2000个以上。水、气等环境质量保持在全省第一方阵。让日照的天更蓝、山更绿、水更清、城市更文明。

实现上述目标，必须立足日照实际，深入贯彻创新、协调、绿色、开放、共享发展理念，大力实施“五大战略”。

实施“生态立市”战略。树立“绿水青山就是金山银山”理念，深入实施“林水会战”，坚持生产和生活两头入手、治理和保护两手齐抓、约束和激励两端发力，建设山青水秀美丽日照。未来五年，完成造林45万亩，创建为国家森林城市。完成中小河流治理1000公里，水土流失治理率达到87%以上，重点河流、水库和水生态持续改善。万元地区生产总值、万元工业增加值用水量分别下降13%。万元地区生产总值能耗下降18%以上，完成主要污染物总量减排任务。“蓝天白云、繁星闪烁”天数每年保持在300天以上。

实施“工业强市”战略。坚持以“扩量提质增效”为目标，做足“建链、强链、补链”文章，推进钢铁、石化、汽车及零部件、粮油食品四大主导产业提升，加快浆纸、建材、能源、纺织服装四大传统产业转型，实施“互联网+”行动、智能制造工程和领军型、成长型企业培育工程，打造山东半岛重要临港产业基地。未来五年，培育4个千亿级产业、10家百亿企业集团、100家“专精特新”小巨人企业，高新技术产业产值占比赶上全省平均水平。

实施“旅游富市”战略。充分发挥海、山、古、林、泉资源优势，实施全域旅游规划，做足“旅游+”文章，推进智慧旅游城市、旅游集散中心、旅游休闲街区等六大工程建设，加强旅游服务规范化、标准化建设，规范整顿旅游市场，构建“海山联动、城乡互动、多业融合、全域发展”的新格局，打造滨海生态宜居旅游城市。到2021年，全市旅游消费总额达到630亿元，创建为国家全域旅游示范区。

实施“开放活市”战略。强化全方位开放意识，用好用活国家自贸区政策，发挥中澳（日照）产业园、综合保税区等平台作用，主动融入“一带一路”国家战略。突出“双港带动、多园支撑、三外融合”，坚持内外需协调、“引进来”与“走出去”并重，提高双向开放水平。到2021年，全市进出口总值突破1000亿元，其中出口年均增长6%以上，累计利用外资30亿美元。

实施“人才兴市”战略。坚持人才优先发展，深入实施“专项招才引智行动”，统筹融合人才与产业、平台、载体建设，深化人才发展体制机制改革，支持驻日照高校争创“双一流”，把日照建设成为各类人才干事有舞台、发展有空间、梦想能实现的人才高地。未来五年，新引进高层次创业人才100人以上、创新团队200个以上、创新人才2000人以上，新引进院士30人、千人计划专家50人、博士毕业生500人以上。

三、2017年政府工作

2017年，是新一届政府开局之年。贯彻落实好市第十三次党代会精神，做好今年各项工作，意义重大，影响深远。主要预期目标是：地区生产总值增长9%左右，一般公共预算收入同口径增长10%左右；固定资产投资增长15%左右，社会消费品零售总额增长9%左右，进出口总值增长3%左右；城乡居民人均可支配收入分别增长8.5%和9%左右；城镇登记失业率控制在3.5%以内；居民消费价格涨幅控制在3%左右；完成省下达的节能减排降碳约束性目标。

（一）保持发展定力，大力振兴实体经济。坚持稳中求进工作总基调，严格落实“三去一降一补”，在“稳”的基础上，多积蓄“进”的力量，努力跑出一个高于全省平均水平的“日照加速度”。

坚定不移推进“突破园区、聚力招引”头号工程。一是提升园区承载能力。推动《日照园区整体发展规划（2016-2030）》全面落地，统筹各园区空间和产业布局，促进园区错位发展、特色发展。创新园区投入机制，推动市级国有融资平台与重点园区开展融资合作，提高园区融资建设能力。完善园区水、电、路、气、暖等基础设施，统筹建设研发设计、检验检测、成果转化、创业风投等公共服务平台，下大力气补齐园区基础设施欠账。二是壮大园区经济实力。对钢铁、石化、汽车、旅游、航空、物流、粮油等重点产业，组建专门班子，抓推进、抓落实。山钢日照精品钢、日钢ESP生产线、清洁能源综合利用等213个市级重点项目，年内完成投资550亿元以上。分类制定优惠政策，推广“租金换股权”等模式，支持入园企业发展。对接国家和省产业政策包、重大工程包，争取更多的资金和项目，投向园区基础设施、科技创新领域。举全市之力支持日照高新区，赋予市级经济管理权限，推动高新区加快升级步伐。三是强化“三招三引”。创新产业招商、专业招商、资本招商等市场化招商方式，抓好熟地储备、标准化厂房等要素供给，保障项目落地。深度对接世界500强、中国500强和行业领军企业，加大招商引资力度，有针对性地引进高层次人才和团队。年内，新引进过5000万元的产业项目200个以上，过10亿元的20个以上，实际到位资金300亿元以上；新引进高层次创业人才20人、创新团队40个、创新人才400人，引进院士6人、千人计划专家10人、博士毕业生70人以上。

坚定不移把工业作为强市之本。一是助推企业转型升级。发挥产业引导基金杠杆作用，实施主导产业提升、传统产业转型、新兴产业倍增“三大工程”，分类制定转型升级方案。市级“百项重点工业”项目，完成投资140亿元以上。亚太森博溶解浆等技改项目，完成投资360亿元以上。二是发展壮大制造业。对接“中国制造2025”，建设工业云、大数据支撑平台，重点推进山钢、日钢、金禾生化等10项“机器换人”项目，推进裕鑫动力等30项“两化”融合项目。办好中国机器人竞赛系列活动。年内，全市净增规模以上工业企业50家以上。三是优化服务环境。落实减轻企业负担“五降一优”42条措施。加大金融创新力度，推动金融支持实体经济发展。完成重点企业风险化解任务。鼓励企业挂牌上市，年内在新三板和区域性股权交易市场挂牌企业20家以上，直接融资150亿元以上。四是实施质量强市和品牌战略。全面加强质量管理，强化商标、品牌、标准引领，创建全国知名品牌示范区。支持企业加强自主品牌建设，申报马德里国际商标注册，争创中国驰名著名商标，提高产品市场竞争力。鼓励企业发扬“工匠”精神，增强企业研发设计能力，瞄准行业标杆，培育百年老店。

坚定不移打造以旅游业为龙头的现代服务业。一是培育发展旅游业。加强整体策划，统筹资源开发，规范整顿市场，创建国家全域旅游示范区。依托“仙境海岸”，对接“东方圣地”“平安泰山”等文化旅游目的地，在“好客山东”旅游大格局中，把“阳光海岸、活力日照”品牌叫响。发挥生态资源优势，吸引国内外大型旅游企业和战略投资者，打造一批多样化、个性化的旅游产品。做足“旅游+”文章，推动旅游与农业、工业，与文化、体育、航空、会展、养生等产业融合发展。突出“一带、三区、两山”等重点区域，抓好东方太阳城等122个旅游项目建设，年内完成投资100亿元以上。整合海岸线资源，全域规划，系统开发，高标准建设“中国（日照）阳光海岸带”。筹办世界太阳文化论坛。推动山海天旅游度假区创建国家级旅游度假区。全年接待游客突破4500万人次，旅游消费总额达到360亿元以上。二是壮大临港物流业。发挥港口物流枢纽作用，积极培育物流园区和龙头企业，打造大宗商品交易中心、公路港等物流平台，建设区域性国际航运物流中心。全社会物流总额达到5200亿元。三是繁荣商贸流通业。结合商业地产去库存，完善商业网点规划布局，改造老城区商圈，提升新市区商圈。高标准完成青岛路城市设计，规划建设国际会展中心、丁肇中科技馆、大剧院，打造集商业、旅游、会展、总部、文化创意、健康养生等功能于一体的城市商业中心。四是培育楼宇经济。吸引大企业大集团在日照设立区域总部，培育一批金融楼、商务楼、科研楼、中介服务楼、现代物流楼等特色楼宇。加快财富中心建设。支持日照银行跨区域扩张发展。加大基金产业发展扶持力度，规划建设基金小镇。全市各类基金规模达到200亿元以上。

坚定不移推进农业现代化。深化农业供给侧结构性改革，提高农业供给质量，促进农村一二三产融合发展。抓好30万亩粮食高产创建示范方建设。实施23项农田灌溉工程，新增节水灌溉面积14.1万亩。改造省级农业标准化生产基地62个，新发展生态循环示范基地3000亩以上。推进万泽丰、顺风阳光等海洋牧场建设，抓好冷水团养殖项目，积极培育“海上粮仓”。加强省级农业科技园建设，争创省级农业高新区。健全农产品质量安全体系，提高市县两级农检中心检测能力。加强农业品牌建设，培育农产品知名品牌10个以上，认证“三品一标”30个以上。保护“日照绿茶”品质品牌，实行母子商标管理制度，提升品牌价值，努力“让日照绿茶卖得更好”。

坚定不移加快民营经济发展。大力推进“大众创业、万众创新”，把激发民间投资作为振兴实体经济的重要支撑。完善市场准入、信贷支持、财政奖补等优惠政策，充分调动全社会创业创新积极性。加强对口服务，对市场占有率在全市50%、全省30%、全国10%以上的民营企业，给予重点扶持，促进民营企业向“专精特新”发展，培育更多行业领军企业、小巨人、“独角兽”。加强与各地日照商会的沟通合作，搭建政企沟通和企业服务平台，鼓励引导在外日照籍人士回乡创业。支持民营企业加快改制步伐，建立现代企业制度。加强民营企业家队伍建设，完善激励机制，让民营企业家政治上有荣誉、社会上有地位、经济上得实惠。年内，民营经济增加值增长20%以上，新增民营企业5000家以上。

（二）强化统筹协调，着力推进城乡一体发展。坚持协调发展理念，把城市和农村作为一个整体来谋划，实现基础设施统筹规划，公共服务全域覆盖，努力让城乡居民共享改革发展成果。

发展壮大县域经济。完善县域经济发展扶持政策，健全财政投入、国资收益、基金增值等滚动投入机制。推进区县投融资平台混改，释放县域经济发展活力。制定县域经济分类考核办法，支持东港区重点发展高新技术、航空装备和现代服务业，推动空港新区、太阳文化旅游度假区开发建设；岚山区重点发展钢铁、石化等临港产业，加快中澳（日照）产业园建设；莒县重点发展机械制造、石化、文化旅游和现代农业；五莲县重点发展全域旅游、生态产业、汽车及农用装备产业。

切实抓好“三农”工作。围绕农业增效、农民增收、农村增绿，把农业农村作为财政支出优先保障领域，确保农业农村投入稳定增长。完善“三权分置”办法，落实集体所有权，稳定农户承包权，放活土地经营权。推进农村集体产权制度改革，年内再完成260个村居集体产权股份合作试点。探索推行“房地票”制度，实行农民宅基地有偿退出机制。实施新型农民培训计划。完善农民“双创”扶持政策，支持农民工返乡创业。落实强农惠农政策，健全农民增收长效机制。培育休闲农业、乡村旅游等新业态，推动“产区变景区、民房变客房、产品变礼品”，提高农民经营性收入比重。

积极培育特色小镇。加快新型城镇化建设，稳步推进乡改镇、镇改街道，对重点示范镇实行扩权强镇，推进农业转移人口市民化。完善用地、财政、金融等政策，重点培育省级重点示范镇夏庄镇以及后村航空小镇、潮河白鹭湾艺游小镇2个省级特色小镇，打造东夷小镇、陈疃莓香、涛雒文化旅游、巨峰茶香慢城、虎山钢铁、中楼橡胶、浮来山旅游、刘官庄塑料、管帅温泉养生等一批特色产业小镇，推动“产、城、人、文”融合发展。年内新争创2个省级特色小镇，打造省级达标美丽乡村500个以上。

大力推进城乡一体化。围绕交通一体化，编制交通基础设施五年规划，投资45.5亿元，实施313省道莒县段改建等10个国省道项目；投资8.2亿元，完善莒北、莲西、港西、岚西北路网，完成13条县乡公路升级改造工程；投资2.5亿元，实施农村公路网化示范县工程，改造村级公路412公里。围绕公交一体化，实行城乡客运班线公交化运营，实现主城区镇镇通公交车，开通区县间公交线路。围绕环卫一体化，建立垃圾分类收集处理系统，构建覆盖城乡的市场化、专业化环卫保洁机制。围绕供气一体化，实施天然气镇镇通工程，实现乡镇供气全覆盖。围绕供水一体化，实施农村自来水提质改造，实现农村供水城市化。围绕供电一体化，加快12项输变电、740项中低压配电网工程建设，实现城乡供电同质同价。统筹推进城乡教育、卫生、文化、体育、健康养生等公共服务一体化。

（三）立足市域一体，全面提高城市规划建设管理水平。强化全局意识，树立系统思维，转变城市发展方式，着力解决“城市病”等突出问题，努力提升城市档次和品位。

坚持高水平规划。按照“生态、宜居、旅游”的城市定位，坚持以“海”为中心，做好“城海互动、向海发展”的文章，用世界眼光和国际标准谋划城市发展。完成《日照市城市总体规划（2015-2030年）》《涛雒太阳文化旅游区总体规划》等总规修编。完成日照火车站及周边区域城市设计等4项详规编制。完成公共交通及轨道线网等11项专项规划编制。实施莒县8项城区规划修编。完善五莲县东部新城区规划修编。

坚持高标准建设。加大老城区、石臼片区改造提升力度。开工建设日照火车站及周边综合服务设施。完成奎山综合客运站主体工程。打通金阳路、华阳路等32条断头路，改扩建16条城市道路。建设改造市民公园（铁路公园）、海曲公园、兴海公园、体育公园，以及岚山阿掖山森林公园、莒县沭河公园、五莲中心公园等市民休闲公园。提升城市主要出入口以及52条城市道路绿化水平。新建黄海一路、天津路、祥博社区、金阳路、五莲路、原日照六中6处街头绿地。整治老旧小区55个、173.9万平方米。拆迁改造72个城中村、2.4万户房屋，建设棚改安置房5.7万套。开发利用城市地下空间，推进综合管廊、人防工程和海绵城市建设。莒县改造建设浮来路等21条道路，完成棚户区改造2.23万套，推进莒国古城、沭东新城建设。五莲县实施山阳河治理等103项重点工程，改善老城区、东部新城区、北部工业新区环境，创建国家园林县城。

坚持精细化管理。加快城市管理立法，深化综合行政执法改革，创新城市治理方式，实行城市管理事权和责任清单式管理，提高城市管理和服务水平。实施文明创建工程。深入推进创城突出问题综合整治，加大环境卫生、公共秩序和社会风尚整治力度，抓好道路沿线环境和城乡违建专项治理，推行“楼长制”“街长制”，完善城乡社区服务功能，争创全国文明城市。实施城市亮化工程。对主城区和县城的重点区域、重要路段，进行亮化提升设计和改造，打造具有滨海特色的城市亮化景观。实施便民出行工程。完成公共交通基础设施规划编制，建设智慧停车公共服务平台，新增停车位2万个以上。建设山东路、青岛路、烟台路3条公交专用道，改造提升100处公交候车亭。加快城市慢行系统建设，投放200辆电动租赁汽车，新增自行车站点100个、投放公共自行车2000辆。对主城区交通信号灯进行配时优化，对100处路口红绿灯实行联网联控，努力让群众出行更顺畅、更安全。

（四）持续保障和改善民生，让人民群众生活更美好。时刻把群众的安危冷暖放在心上，努力让改革发展成果更多更公平地惠及全市人民。

坚决打赢脱贫攻坚战。突出莒北、莲西、港西、岚西北四个重点区域，继续实施产业就业、基础设施建设、易地搬迁等十项精准扶贫行动，确保稳定实现“两不愁、三保障”。完善教育扶贫、医疗扶贫、孝德养老等政策措施，着力解决贫困人口上学难、看病难、养老难等问题。实行低保扩面兜底，确保应保尽保。将农村贫困人口全部纳入重特大疾病医疗救助范围。完善扶贫长效机制，年内再脱贫1万人以上，基本完成脱贫攻坚任务。

坚持教育优先发展。完成“全面改薄”“大班额”和学前教育二期行动计划。投资10.79亿元，新建、改扩建学校50所、幼儿园37处。深化中小学校长职级制改革、教师县管校聘改革，实施乡村教师支持计划，新招聘教师1000名。加快发展现代职业教育，培养日照紧缺的技术技能人才。支持日照职业技术学院创建国家优质高职院校，支持日照技师学院争创全国示范性技师学院，支持山东外国语职业学院、日照海事专修学院提升办学层次。为驻日照高校创造良好发展环境，促进校地双方共同发展。重视发展社区教育、特殊教育，加快发展民办教育。

积极扩大就业创业。广开就业渠道，开展“订单式”培训，强化急需高技能人才培养。鼓励以创业带动就业，统筹推进市、县、乡三级创业服务平台建设，新增市级创业孵化示范基地5家以上。鼓励支持高校毕业生、妇女等各类群体就业创业。做好零就业家庭就业援助和社会保障托底。完成农民工职业技能提升、权益保障、公共服务“三年行动计划”，开展保障农民工工资支付专项行动。新增城镇就业2.6万人，完成职业技能培训1.3万人。

提高卫生健康水平。推动“健康城市”建设。加快城市公立医院改革发展，统筹规划建设公立医院基础设施，完善周边配套服务设施。加强基层卫生工作，推进分级诊疗，提高综合服务能力。支持民营医院发展，建成日照心脏病医院等高水平专科医院。坚持中西医并重，支持中医药重点专科发展。推动全民健身与全民健康深度融合，建设便民惠民体育公共服务体系。统筹建设全民健身场所，新建全民健身点100处。支持国家足球学院、篮球学院建设。发挥海滨山岳、水上运动优势，办好国际马拉松、国际啦啦操、国际太极拳等系列赛事活动，打造活力之城、运动之城、健康之城。

完善社会保障体系。实施全民参保登记计划，深化养老保险制度改革，健全失地农民社会保障体系。城乡居民低保标准分别提高到520元/月、340元/月。统筹社会保险和社会救助制度，健全扶老助残救孤服务体系。农村贫困人口大病保险，年度最高补偿额提高到50万元。加强对农村留守儿童、老人的关爱服务。开展莒县、五莲县民生综合保险试点。规范家政服务等中介服务机构。推进公办养老机构公建民营。

优化公共文化服务。实施“千百重点文化工程”，加强基层综合文化服务中心建设，村级达标率达到90%以上。设立文化产业发展基金，培育文化创意产业。深入开展太阳文化等特色文化研究。办好第八届中国少年儿童合唱节。完善提升市美术馆、文化馆、图书馆、博物馆功能，推进各级国家综合档案馆建设。倡导“德润港城•善行日照”，开展移风易俗乡村文明行动，培养向上向善的良好社会风尚。

保持社会和谐稳定。深化“平安日照”建设，争创“长安杯”。加强政法综治基层基础建设，完善立体化、信息化治安防控体系。建立“一村一法律顾问”“一村一警务助理”制度。高度重视群众来信来访，健全社会矛盾排查、预警、调处机制，多元化解矛盾纠纷，及时回应群众诉求。加强社会诚信建设，依法严厉打击逃废银行债务等失信行为。支持人民法院解决执行难问题。落实“七五”普法规划，建设法治日照。健全可防可控安全生产“1+4”多重预防体系。强化食品药品安全监管，争创省级食品安全城市。推进军民融合深度发展，支持驻军、武警部队建设，争创全国双拥模范城“六连冠”。做好援疆和扶贫协作重庆市黔江区工作。

（五）守住生态底线，建设山青水秀美丽日照。加强生态文明建设，守护绿水青山，留住碧海蓝天，努力让人民群众生活得更安全、更健康。

抓好生态修复。深入推进“林水会战”，投资35.8亿元，造林15万亩，建设农田水利工程1621项，治理河道155公里。倡导全民植树造林，加快“林中花海”“山间竹海”建设，争创国家森林城市。完善现代水网，实施水系连通、中水回用、异地调水、海水淡化等工程，建立覆盖全市的治水管水“生态链”。实行最严格的水资源管理制度，实施日照水库环库生态、青峰岭水库生态湿地工程。实施海洋规划、岸线修复、综合管控工程，改善海洋生态，打造日照国家海洋公园、国家级海洋生态文明建设示范区、国家级生态保护与建设示范区。

抓好生态治理。全面推行“河长制”，大力实施“清清河流”行动，完成莒县马沟河、五莲县袁公河等32条河流治理工程。完善“治、用、保”治污体系。完成禁养区全部畜禽养殖转产搬迁，实现工业园区污水集中处理，省控重点断面水质合格率达到80%以上。加大大气污染防治力度，抓好工业污染、施工扬尘、燃煤锅炉整治，空气质量稳定保持在全省第一方阵。加大土壤污染防治力度，抓好省级生态循环示范基地建设，改善土壤环境质量，保障农产品和人居环境安全。

抓好生态保护。合理控制城市开发强度，严守生态红线，加大生态敏感区保护力度，建立重点生态功能区补偿机制，建设绿色城市。严格执行项目用地、用水、环保、安全等准入标准，实施钢铁脱硫脱硝等重点减排工程，完成制浆造纸等重点行业清洁化改造，建设绿色企业。加强生态价值观教育，倡导绿色生产方式、生活方式和消费方式，严格落实生态环境损害责任追究制度，建设绿色家园。

（六）深化改革创新开放，激发日照发展更大活力。革除一切妨碍发展的思想观念和体制弊端，强化改革引领、创新驱动、开放支撑，努力为日照更好更快发展注入不竭动力。

向改革要红利。深化国企改革，完善运营监管、激励约束机制，建立职业经理人制度，实行严格的国有企业内部监管和出资人监管，提高国有资产资本化、市场化、证券化水平。深化投融资改革，推行政府购买服务和PPP投资模式。发挥市和区县国有投融资平台作用，全年融资360亿元以上。深化“放管服”改革，建立行政权力精简下放常态化机制，推行“串改并”“互联网+政务服务”，全面实施建设项目区域化评估评审。完善“六证合一、一照一码”企业登记制度，强化事中事后监管，让失信企业“一处违法、处处受限”。深化社保制度改革，完善职工和城乡居民大病保险制度，推进基本医疗保险和生育保险并轨运行。

向创新要动力。开展创新型城市建设。突出企业创新主体地位，实施高新技术企业、知识产权企业培育工程。建立“政府搭台、企业主导、科研院校为依托”的产学研体系，重点建设钢铁研究院、通用航空技术研究院等10个市级产业技术创新研究院，培育钢铁、电子信息、高端装备制造、航空产业等创新型产业集群。规划建设市科技创新综合体。新建10家以上国家和省级孵化器、众创空间、星创天地，建成30个省级以上科技研发服务平台。发挥市人才创新发展院、企业发展研究院的作用，建立高层次人才发展俱乐部，加快建设人才公寓，实施“人才安居工程”。年内，新增高新技术企业40家以上，引进转化国内外先进技术成果100项以上，新增技术创新项目120项以上，市级以上企业技术中心20家以上。

向开放要空间。主动对接周边城市，在规划衔接、产业合作、基础设施互联互通等领域，建立广泛的城市合作伙伴关系。依托瓦日铁路、鲁南高铁等大通道，扩大与中西部经济板块的开放合作。加强与欧美、澳大利亚及周边国家和地区的交流合作，深化产业对接、经贸往来、人文交流。加快空港口岸开放，启动机场扩建工程，建设区县通用机场，旅客吞吐量达到70万人次。加快中澳（日照）产业园建设，推进双边经贸园区合作发展。争取获批设立日照综合保税区，形成开放型经济新优势。推进省级跨境电子商务综合试验区建设，深化与阿里、京东等知名电商合作，推动特色农产品、优质工业品上行。跨境电商出口增长30%以上。

突出抓好日照港改革发展工作。优化港城空间布局，推进“南散北集”，建设月亮湾工程，实现退港还海。科学规划港口集疏运体系，推进岚山港区深水航道二期等10项重点工程，完成石臼港区北京路上海路畅通工程、深圳路东延工程，启动改造岚山港区南疏港路。深化通关流程改革，提高通关效率。推动港口转型升级、创新发展，打造诚信港口、智慧港口、高效港口、绿色港口。从日照港集团案件暴露出的失控漏管、资产流失、体制僵化等问题入手，强化监督管理，深化内部改革，优化治理结构，努力实现由“大港”向“强港”跨越。

四、切实加强政府自身建设

进一步增强政治意识、大局意识、核心意识、看齐意识，对标市委关于讲政治、讲担当、讲效能、讲学习、讲协调、讲纪律的“六讲”要求，恪尽职守、永不懈怠，全面提升政府履职能力和服务水平。

（一）坚持依法行政，建设法治政府。全面落实法治政府建设实施纲要，运用法治思维和法治方式推进政府工作。坚持把公众参与、专家论证、风险评估、合法性审查、集体研究决策，作为重大行政决策的必经程序，发挥政府法律顾问和社会智库辅助决策作用，健全依法科学民主决策机制。完善“四张清单、一个平台”行政权力运行体系，以公开为常态、不公开为例外，全面推进政务公开，让行政权力始终在阳光下运行。自觉接受人大、政协监督，定期听取民主党派、无党派人士和人民团体的意见建议。完善政府新闻发言人和突发事件信息发布制度，畅通政府与公众互动渠道，保障人民群众知情权、参与权、监督权。

（二）坚持实干担当，建设责任政府。教育引导政府系统各级干部深入学习以习近平同志为核心的党中央治国理政新理念新思想新战略，学习经济、文化、生态以及法律、科技、金融等方面专业知识，提高行政能力，克服本领恐慌。持续推动政风转变，大兴调查研究之风，多向群众学习，向实践学习，提高政府决策质量。发扬苦干实干作风和“钉钉子”精神，敢于直面矛盾、破解难题。健全督查问责机制，坚决整肃庸政、懒政、怠政行为，形成勇于担当、勤勉尽责的工作氛围。完善正向激励机制，坚决杜绝“劣币驱逐良币”的逆淘汰现象。

（三）坚持忠诚为民，建设服务政府。始终把保障和改善民生作为政府工作的出发点和落脚点，认真研究解决群众关注的热点难点问题。坚持问政于民、问需于民、问计于民，完善重大行政决策网络问政、公开听证制度，定期听取各界人士意见建议。推进信息化与政府管理融合，优化工作流程，提高服务效率，坚决砍掉各类无谓证明和繁琐手续，真正实现让数据多跑路、群众少跑腿。构建“亲”“清”新型政商关系，完善与企业、商会协会联系沟通机制，为企业发展创造宽松的外部环境。

（四）坚持正风肃纪，建设廉洁政府。始终把纪律和规矩挺在前面，认真贯彻党内政治生活准则和党内监督条例，严格落实政府系统全面从严治党“两个责任”，做到干部清正、政府清廉、政治清明。坚持开正门、堵旁门、关后门，破除各种形式“潜规则”， 最大限度减少公务人员自由裁量权，从源头上防止设租寻租和利益输送。自觉接受社会各界监督，坚决纠正损害群众利益的不正之风。深刻汲取日照港集团等案件的教训，加强对各级领导干部的教育监督，保持反腐败高压态势，构建不敢腐、不能腐、不想腐的有效机制，营造风清气正、干事创业的良好政治生态。

各位代表！蓝图已绘就，奋进正当时。让我们紧密团结在以习近平同志为核心的党中央周围，在省委、省政府和市委的坚强领导下，继续发扬事不避难、勇于担当、苦干实干的精神，不忘初心、继续前进，撸起袖子加油干，为建设美丽富饶、生态宜居、充满活力的新日照而努力奋斗！
